# Vitamin D and Chronic Urticaria: A Review of Potential Mechanisms, Clinical Evidence, and Future Directions

**DOI:** 10.1155/drp/1929442

**Published:** 2026-10-21

**Authors:** Chang Qin, Yuqi Wang, Ye Zou, Dongsheng Zhong, Xiaojie Ding

**Affiliations:** ^1^ Affiliated Hospital of North Sichuan Medical College Nanchong Sichuan China hospital-nsmc.com.cn

**Keywords:** chronic urticaria, immunology, therapeutics, threshold model, vitamin D

## Abstract

Vitamin D has long been recognized for its essential role in calcium and phosphorus homeostasis and skeletal maintenance. Over the past few decades, however, accumulating evidence has uncovered its pivotal nonskeletal function in orchestrating immune responses, sparking considerable interest in its association with various autoimmune disorders and inflammatory skin diseases. Chronic urticaria (CU) is a common skin disease with a complex etiology, and immune dysfunction is closely implicated in its pathogenesis. This narrative review systematically summarizes the epidemiological evidence, potential mechanisms of action, and clinical intervention studies regarding the relationship between vitamin D and CU. The available evidence, however, remains heterogeneous, and causality has not been firmly established owing to the predominance of cross‐sectional designs and the presence of multiple confounding factors. Although most interventional studies report positive effects of vitamin D supplementation as an adjunct to standard therapy, findings are conflicting because of considerable heterogeneity in dosing regimens, treatment duration, and baseline vitamin D status. Based on the reviewed evidence, we propose a “threshold model,” which posits that vitamin D modulates the immune activation threshold rather than directly triggering disease. The deficiency of vitamin D lowers this threshold and increases CU susceptibility, whereas the clinical onset of CU requires additional triggering factors such as infections or autoantibodies. This model remains a theoretical framework requiring further clinical and mechanistic validation. This narrative review aims to provide an integrated perspective for understanding the complex relationship between vitamin D and CU and to offer a theoretical basis for the development of comprehensive treatment strategies.

## 1. Introduction

Definition of chronic urticaria (CU): CU is a common skin condition characterized by recurrent, itchy wheals lasting more than 6 weeks [1]. CU has a complex pathogenesis, is difficult to treat, and is prone to recurrence. Conventional antihistamine therapy is ineffective for some patients, highlighting the necessity of exploring novel therapeutic targets and intervention strategies.

Definition of vitamin D: Vitamin D is an essential fat‐soluble vitamin for humans, existing primarily in two forms: vitamin *D*_2_ (ergocalciferol) and vitamin *D*_3_ (cholecalciferol) [2]. After entering the bloodstream through intestinal absorption or skin synthesis, vitamin D is converted in the liver to 25‐hydroxyvitamin D [25(OH)D] and subsequently in the kidneys to 1,25‐dihydroxyvitamin D [1,25(OH)_2_D] [3]. Beyond its classical role in maintaining calcium and phosphorus homeostasis, recent studies have revealed that vitamin D plays significant roles in inhibiting tumor progression, protecting the cardiovascular system, and regulating autoimmune responses [4].

A growing body of evidence suggests a close association between vitamin D and CU, and some studies have reported that vitamin D supplementation may be linked to improvements in symptoms and quality of life in patients with CU. However, these results are inconsistent across studies due to significant methodological heterogeneity, and a definitive causal relationship has not been validated. This article systematically reviews the epidemiological, mechanistic, and clinical evidence linking vitamin D to CU and evaluates its potential as an adjunctive therapeutic approach, aiming to provide a balanced overview for clinical practice.

Given that most available evidence pertains to chronic spontaneous urticaria (CSU), the predominant subtype of CU, this review primarily focuses on CSU while also discussing findings relevant to CU more broadly.

This is a narrative review. We searched PubMed, Web of Science, and Cochrane Library for articles published up to December 2025 using the keywords: “vitamin D,” “chronic urticaria,” “25‐hydroxyvitamin D,” “supplementation,” “mast cell,” “mechanism,” “Vitamin D Deficiency,” and “vitamin D receptor.” We included original research, systematic reviews, meta‐analyses, case–control studies, and cross‐sectional studies. We also manually searched the bibliographies of relevant articles for additional references. Given the narrative nature of this review, we did not perform a formal quality assessment or quantitative synthesis.

## 2. Epidemiological Association Between Vitamin D Deficiency and CU

Numerous epidemiological investigations have explored differences in vitamin D levels between patients with CU and healthy individuals, as well as the association between vitamin D levels and disease severity. However, the quality of this evidence and its clinical significance still require careful evaluation.

### 2.1. Significantly Higher Prevalence of Vitamin D Deficiency in CU Patients

Cross‐sectional studies consistently report a higher prevalence of vitamin D deficiency and lower serum 25(OH)D concentrations in CU patients. In a key study, Rather et al. reported that approximately 79% of patients with CSU, the predominant CU subtype, had vitamin D insufficiency or deficiency, a rate more than twice the 33.45% observed in the healthy control group [5]. In line with these findings, a 2021 meta‐analysis further quantified this disparity, revealing a weighted mean difference of −9.35 ng/mL in serum 25(OH)D levels between CU patients and controls, with the largest reductions seen in adults with CU and CSU [6].

These findings also highlight a broader public health concern: Even among healthy control groups, vitamin D insufficiency is remarkably common—for example, 33.45% in the study by Rather et al. [5]. This underscores that vitamin D insufficiency and deficiency are not unique to CU patients but constitute a prevalent public health problem worldwide.

### 2.2. Association Between Vitamin D Deficiency and Disease Severity

The 7‐day Urticaria Activity Score (UAS7) is a validated core instrument for assessing disease severity and activity in patients with CU. Several studies have reported a significant inverse correlation between serum vitamin D levels and UAS7 scores, implying that lower vitamin D levels are associated with greater CU disease activity and elevated systemic inflammation [5–7]. Conversely, other studies have failed to detect a statistically significant relationship between vitamin D status and CU disease activity measured by UAS7. For example, Tat et al. observed no meaningful correlation between vitamin D levels and disease activity in patients with CSU [8]. These discrepancies suggest that the relationship between vitamin D and CU is more complex than a simple linear correlation. Rather than directly modulating disease activity, vitamin D primarily affects disease susceptibility rather than directly regulating ongoing inflammatory responses, mediating CU pathogenesis via indirect immunomodulatory mechanisms.

Vitamin D levels have also been associated with pruritus severity, which is one of the most distressing symptoms in CU. In terms of patient‐reported outcomes, several controlled interventional trials have further demonstrated that vitamin D supplementation confers dual clinical benefits: It reduces core CU symptoms and significantly improves dermatology‐specific quality of life, as measured by the Dermatology Life Quality Index (DLQI) [9, 10].

### 2.3. Critical Evaluation

Although epidemiological data consistently show reduced serum vitamin D in patients with CU, several critical limitations must be considered. First, vitamin D status is significantly influenced by external and environmental factors, including geographical latitude, seasonal variation, and daily sun exposure, all of which are well‐established determinants of circulating 25(OH)D levels [6, 11–13]. Second, a range of intrinsic and lifestyle‐related confounding factors, such as obesity, baseline systemic inflammation, and dietary patterns, also exert independent effects on vitamin D synthesis and metabolism [13–15]. Importantly, many studies do not adequately adjust for these variables. Consequently, the observed differences in vitamin D levels between CU patients and healthy controls may be partly attributable to these external factors rather than representing a direct consequence of CU itself. The implications of these confounding factors for future study design and clinical trial interpretation are discussed further in the Discussion.

## 3. Potential Mechanisms by Which Vitamin D Regulates the Immune System and Influences CU

Vitamin D exerts potent and multitarget immunomodulatory effects, and accumulating evidence has underscored its important role in the pathogenesis of CU. The immunomodulatory functions of vitamin D have been increasingly recognized across various allergic conditions, including atopic dermatitis and allergic rhinitis [16]. The underlying mechanisms are largely mediated by the vitamin D receptor (VDR) signaling pathway, which directly regulates mast cell activity and indirectly influences T cells, B cells, and systemic immune homeostasis. These molecular and cellular mechanisms are detailed in the following subsections.

### 3.1. Expression and Signal Transduction of the VDR

The biological effects of vitamin D are predominantly mediated via binding to the VDR, a well‐characterized nuclear receptor [17]. As a classical member of the nuclear receptor superfamily, VDR exerts its transcriptional regulatory functions through a conserved signaling cascade. Upon ligand binding, VDR forms a stable heterodimer with the retinoid *X* receptor (RXR), and this complex subsequently binds to vitamin D response elements (VDREs) in the promoter regions of target genes, thereby modulating the transcription of immune‐related genes and fine‐tuning systemic immune responses [18].

### 3.2. Direct Regulation of Mast Cells by Vitamin D

In CU, mast cell activation releases inflammatory mediators that directly trigger wheal formation and pruritus [19]. Vitamin D exerts multiple regulatory effects on mast cells. On the one hand, it induces mast cell apoptosis and inhibits proliferation via VDR [20]; supplementation with calcitriol can upregulate VDR expression in mast cells, thereby preventing their spontaneous activation in the absence of stimuli [21]; on the other hand, it inhibits IgE‐mediated mast cell activation, directly reducing the release of proinflammatory and vasodilatory mediators, and can interrupt the PI3 K/Akt/p38 MAPK/HIF‐1*α* signaling axis to inhibit VEGF production by mast cells [22, 23].

### 3.3. Indirect Regulation of T Cell Subpopulations by Vitamin D

Although mast cells are the effectors in the final stage of the immune response, vitamin D can also influence the activation state of mast cells indirectly by regulating T‐cell differentiation and function.

#### 3.3.1. Induction of Regulatory T Cells (Tregs)

Vitamin D contributes to both innate and adaptive immunity [24]. It promotes the generation and function of Tregs, facilitates the conversion of CD4^+^ T cells into IL‐17‐secreting Tregs, and increases the secretion of the anti‐inflammatory cytokine IL‐10, thereby reinforcing immune tolerance and attenuating inflammation [4].

#### 3.3.2. Regulation of Th1/Th2 Balance

Vitamin D regulates T‐cell polarization and immune responses through multiple pathways. On the one hand, it promotes the production of antimicrobial agents by target cells, drives T‐cell polarization toward a Th2 phenotype, inhibits Th1 cell proliferation and the secretion of Th1/Th17 proinflammatory cytokines, and induces Th2 cells to produce anti‐inflammatory cytokines [24]; on the other hand, its active form, 1,25(OH)_2_ *D*_3_, reprograms dendritic cells via transcription‐mediated metabolic pathways that enhance glycolysis and oxidative phosphorylation. Consequently, dendritic cells modulate T‐cell behavior, promoting anergy, apoptosis, and a shift from a proinflammatory Th1/Th17 response toward a more tolerogenic Th2/Treg profile [4].

#### 3.3.3. Regulation of the Th17/Treg Balance

Th17 cells, characterized by the secretion of IL‐17, are pivotal in autoimmune diseases. In patients with CU, particularly those with coexisting autoimmune diseases, peripheral blood IL‐17 levels, the proportion of Th17 cells, and the Th17/Treg ratio are elevated [25]. Vitamin D inhibits the differentiation of naive CD4+ T cells into Th1 cells, reduces the production of Th1 and Th17 cytokines, and regulates the development of Th2 and Treg cells. As a potent inhibitor of Th17 differentiation, it reverses the aforementioned immune dysregulation [26].

### 3.4. Effects of Vitamin D on B Cells and Autoantibodies

A subset of CU patients exhibits an autoimmune component, with circulating autoantibodies against the high‐affinity IgE receptor (FcεRI) or against IgE itself [27]. Vitamin D directly modulates B‐cell function by promoting IL‐10 production [28], inhibiting the differentiation and maturation of naive B cells, inducing apoptosis in activated B cells, and reducing IgE synthesis [29]; simultaneously, through Treg enhancement and Th17 suppression, it may indirectly reduce the production of autoantibodies [30]. However, direct evidence for its effect on autoantibody titers in CU patients remains limited and warrants further investigation.

## 4. Recent Advances in Clinical Research on Vitamin D Supplementation for the Treatment of CU

The potential value of vitamin D in the treatment of CU has attracted widespread attention, but the results of clinical studies have not been entirely consistent. This section systematically reviews the existing clinical evidence and analyzes the impact of different study designs, dosing regimens, and patient characteristics on treatment outcomes (Table 1).

**TABLE 1:** Summary of key clinical studies on vitamin D supplementation in CU.

Author, year	Study design	Sample size	Baseline vitamin D status	Intervention	Duration	Key outcomes	Result
Rorie et al., 2014 [31]	RCT	*N* = 42	Not specified	Vit *D*_3_ 4000 IU/d vs. 600 IU/d	12 weeks	Symptom control	Positive (high‐dose superior)
Rasool et al., 2015 [32]	Randomized case–control study	*N* = 147 CSU, 130 controls	CSU patients lower than controls	Vit *D*_3_ 60,000 IU/week	10 weeks	Symptom resolution (5‐D score and VAS)	Positive
Topal et al., 2016 [10]	Prospective case–control study	*N* = 58 CSU, 45 controls	Low	Vit D 300,000 IU/month	3 months	Symptom resolution (UAS4 and CU‐Q2oL)	Positive
Woo et al., 2015 [33]	Retrospective case–control study	*N* = 72 CU, 72 controls	Low in CU	No intervention	N/A	Disease severity (UAS)	Observational: deficiency linked to severity
Ariaee et al., 2017 [34]	RCT	*N* = 20 CSU	Vitamin D deficiency	Vit D 50,000 IU/week	8 weeks	Symptom control (DLQI, USS)	Positive
Mony et al., 2020 [35]	RCT	*N* = 120 CU	Vitamin D deficiency	Vit D 60,000 IU/week	12 weeks	Clinical outcomes (UAS7), inflammation	Positive
Mohamed et al., 2022 [36]	RCT	*N* = 77 CSU, 67 controls	CSU lower than controls	0.25 μg alfacalcidol/d	12 weeks	25 (OH)D levels, symptom control (USA7), inflammation	Positive
Tat et al., 2025 [8]	Observational study	*N* = 105 CSU, 140 controls	CSU lower than controls	No intervention	N/A	Disease severity (UAS7)	No statistically significant relationship between disease severity and vitamin D levels

*Note:* 25(OH)D, 25‐hydroxyvitamin D; CU‐Q2oL, Chronic Urticaria Quality of Life Questionnaire; only studies discussed in the main text are included in this table.

Abbreviations: CU, chronic urticaria; CSU, chronic spontaneous urticaria; DLQI, Dermatology Life Quality Index; N/A, not applicable; QoL, quality of life; RCT, randomized controlled trial; UAS, Urticaria Activity Score; USS, Urticaria Severity Score; VAS, Visual Analog Scale.

### 4.1. Summary of Existing Clinical Studies

#### 4.1.1. Studies With Positive Results: Vitamin D is Effective as an Adjuvant Therapy

Most interventional studies report that vitamin D supplementation confers benefits in patients with CU, especially when added to standard antihistamine therapy. Studies by Rorie et al., Rasool et al., Mohamed et al., Ariaee et al., Topal et al., and Mony et al. all administered vitamin D or its analogs in addition to standard antihistamine therapy. The results demonstrated significant improvements in symptoms, reduced inflammation levels, and enhanced quality of life in patients with CU/CSU, with high‐dose regimens showing superior efficacy compared to low‐dose regimens [10, 31, 32, 34–36].

#### 4.1.2. Studies With Negative Results or No Observed Association

A minority of studies have not confirmed an association between vitamin D levels and CU, or have not observed significant benefits from supplementation. Tat et al. found no clear association between CSU disease severity and vitamin D levels [8]; Vurgun et al. suggested that lower 25(OH)D levels in CSU patients might be a secondary phenomenon rather than a cause of CSU [37]. While some observational studies identified low vitamin D levels in CU patients, they did not conduct supplementation interventions to validate these findings [33].

#### 4.1.3. Studies Focusing Exclusively on Patients With Deficiency

Baseline vitamin D status appears to be a critical stratification factor influencing study outcomes. Studies that specifically enrolled deficient patients tend to show more pronounced clinical improvements. For example, Ariaee et al. and Mony et al. restricted recruitment to CU/CSU patients with documented vitamin D deficiency and reported positive outcomes [34, 35].

### 4.2. Controversies Regarding Dosage and Treatment Duration

#### 4.2.1. Differences in Dosage Regimens

In existing studies, dosing regimens are primarily divided into three categories: (1) daily low‐dose regimens, such as the 4000 IU/day regimen compared with 600 IU/day used by Rorie et al. (2014) [31]; (2) weekly high‐dose regimens, the most common being 60,000 IU/week [35, 36] or 50,000 IU/week [34]; and (3) monthly single‐dose loading regimens, such as 300,000 IU/month [10]. Although convenient, the latter results in significant fluctuations in blood levels.

#### 4.2.2. Impact of Target Serum Levels

The optimal serum 25(OH)D concentration remains debated. Some researchers contend that higher levels are necessary for immunomodulatory effects and recommend maintaining serum 25(OH)D at a minimum of 30 ng/mL (75 nmol/L), with 40–60 ng/mL (100–150 nmol/L) being optimal, to achieve the best overall health benefits of vitamin D [3, 38].

#### 4.2.3. Variations in Treatment Duration

Treatment duration varies considerably across studies, contributing to heterogeneity. A meta‐analysis with subgroup analysis by duration found that topical vitamin *D*_3_ for less than 8 weeks significantly reduced pruritus severity; moderate effects were observed with oral vitamin *D*_3_ within 8–12 weeks, whereas treatment durations of 12–24 weeks failed to yield significant antipruritic effects [39].

### 4.3. New Evidence for Causal Inference: Mendelian Randomization Studies

A two‐sample bidirectional Mendelian randomization study has provided genetic evidence supporting a causal effect of vitamin D on urticaria risk. Reverse Mendelian randomization analysis did not indicate that genetic risk for urticaria influences vitamin D levels, implying a unidirectional relationship (vitamin D may affect urticaria, not the reverse) [40]. However, replication in independent cohorts is essential to confirm these genetic findings.

Overall, although most intervention studies report positive effects, substantial heterogeneity in dosing, duration, and baseline vitamin D status precludes definitive conclusions. A 2025 systematic review comprising 11 studies (910 patients with CU and 581 healthy controls) confirmed this heterogeneity [41]. The strongest positive signals come from studies restricted to deficient patients receiving high‐dose regimens, yet negative findings should not be dismissed.

## 5. Mechanism Integration and Individual Differences: Threshold Models and Genetic Polymorphisms

Vitamin D deficiency probably contributes to the onset or persistence of CU by influencing multiple immune pathways, including the regulation of mast cell function, Treg function, and autoantibody production. These mechanisms are not independent but form a complex regulatory network.

### 5.1. Threshold Model: Vitamin D as a “Regulatory Valve” in Immune Modulation

Based on the integration of the aforementioned mechanisms, this study proposes a threshold model for the pathogenesis of CU. The core concept of this model is that vitamin D is not a “switch” for CU onset but rather a “regulator” of immune system activation.

Low vitamin D lowers the threshold, increasing the likelihood of disease initiation or persistence; however, clinical exacerbations are triggered only when combined with “secondary triggers” such as infections, allergens, and autoantibodies (Figure 1).

**FIGURE 1:**
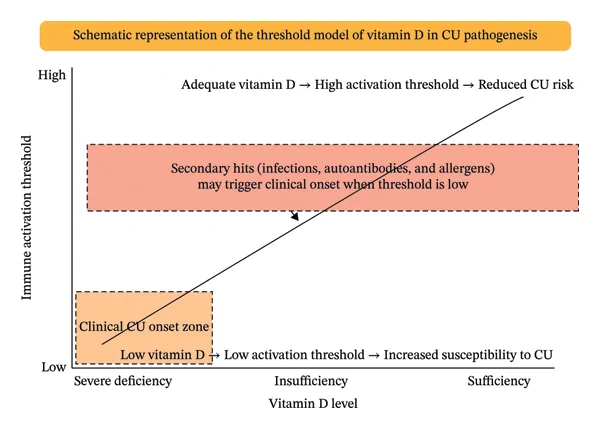
Schematic representation of the threshold model of vitamin D in CU pathogenesis.

It is important to distinguish between components of this model that are supported by current evidence and those that remain hypothetical:

Evidence‐supported components:

1. Vitamin D maintains mast cell stability via VDR‐mediated signaling, reducing spontaneous and IgE‐mediated activation [20–23].
2. Vitamin D promotes Treg differentiation and IL‐10 secretion, bolstering immune tolerance [4, 26].
3. Vitamin D suppresses Th17 differentiation and associated inflammatory responses [25, 26].
4. Vitamin D directly modulates B‐cell function in vitro (promoting IL‐10 production, inhibiting naive B‐cell differentiation and maturation, inducing apoptosis, and reducing IgE synthesis) [28, 29]; it may also indirectly lower autoantibody production via Treg/Th17 modulation [27, 30]. However, direct evidence for these B‐cell effects in patients with CU remains limited.
5. Vitamin D deficiency is epidemiologically associated with higher CU prevalence [5, 6].

Hypothetical components requiring validation:

1. The precise quantitative relationship between serum 25(OH)D levels and the immune activation threshold (i.e., the shape of the dose–response curve) remains undefined.
2. The concept that “secondary hits” such as infections or autoantibodies are required to cross the threshold in vitamin D‐deficient individuals remains speculative and has not been directly tested.
3. The hypothesis that supplementation during disease remission may be more effective than during active flares requires prospective validation.

This model accounts for several clinical observations: why not all individuals with vitamin D deficiency develop CU, why vitamin D supplementation exhibits variable efficacy among CU patients, and why the association between vitamin D and disease severity is not always consistent.

### 5.2. Individual Variability: The Influence of Genetic Polymorphisms in the Vitamin D Pathway

There is significant individual variability in the immunomodulatory effects of vitamin D, and genetic factors play a crucial role in this variability. Not all patients with CU respond equally to vitamin D supplementation, so identifying subgroups likely to benefit is crucial for clinical practice. Studies suggest that patients with autoimmune urticaria, those with extremely low serum 25(OH)D levels (< 10 ng/mL), and carriers of specific VDR genotypes may respond better to vitamin D therapy [42, 43]. Trefilio et al. systematically reviewed genetic variants associated with vitamin D and autoimmune diseases, finding that the variant loci in most studies were concentrated at *Fok*I (rs2228570), *Bsm*I (rs1544410), *Apa*I (rs7975232), and *Taq*I (rs731236) sites within the VDR gene [44]. Polymorphisms in these genes may, to some extent, explain why some patients respond well to supplementation while others do not.

Nevertheless, these genetic findings are preliminary. Clinical application for patient stratification requires prospective validation in adequately powered trials before it can be translated into practice. Several limitations should be noted: Most VDR polymorphism studies have been conducted in specific ethnic groups, and effect sizes vary considerably across studies. Future studies should incorporate genetic stratification in RCT designs to determine whether VDR genotype predicts differential responses to supplementation.

## 6. Discussion and Future Research Directions

The complex relationship between vitamin D and CU has attracted increasing research attention, yet multiple critical research gaps remain unaddressed.

Establishing causality remains a central challenge. Cross‐sectional studies have consistently demonstrated a negative correlation between vitamin D levels and CU activity, but the direction of this association remains unresolved. Vitamin D deficiency could directly promote CU via VDR‐mediated mast cell stabilization through VDR–Lyn kinase interaction and suppression of the PI3K/Akt/p38 MAPK/HIF‐1*α* axis [22, 23], promotion of Treg differentiation and IL‐10 secretion, and suppression of Th17‐mediated inflammation [4, 26]. Conversely, CU itself might lower vitamin D through chronic inflammation‐induced catabolism [45], reduced UVB exposure due to decreased outdoor activity [11, 12], and reduced dietary vitamin D intake [13], with comorbid obesity and autoimmune thyroiditis further compounding the effect [14, 45]. These pathways may operate simultaneously, establishing a bidirectional feedback loop that perpetuates both conditions. This complexity underscores the limitations of cross‐sectional studies and highlights the need for prospective cohort studies and Mendelian randomization analyses to exclude confounding factors and validate the causal relationship. Chai et al. provided genetic evidence for a unidirectional effect of vitamin D on urticaria risk (OR = 0.81, 95% CI: 0.69–0.95) [40], but replication is essential. Future studies should adopt longitudinal designs with repeated measures of vitamin D and disease activity.

Confounding factors influence both vitamin D status and treatment response. Beyond causality, several additional factors may influence both vitamin D status and the response to supplementation in patients with CU, and these should be carefully considered in future research design. Obesity is a well‐established determinant of vitamin D status, as 1.25(OH)D inhibits adipogenesis through actions modulated by VDR; obese patients may require higher supplementation doses to achieve equivalent serum 25(OH)D levels [14]. Sunlight exposure and dietary habits vary considerably across populations and directly affect endogenous vitamin D synthesis and intake, yet few CU studies have adequately controlled for these variables [11–13]. Comorbid autoimmune diseases, especially autoimmune thyroiditis, which affects approximately 25%–30% of CSU patients, may independently influence both vitamin D metabolism and CU disease activity through shared immunopathological mechanisms [27, 45]. Additionally, concurrent medications such as glucocorticoids, which are frequently used in severe CU, may affect vitamin D metabolism via their influence on cytochrome P450 enzymes [4]. These factors may act as important confounders or effect modifiers in observational studies and clinical trials. Future studies should systematically collect and adjust for these variables, and subgroup analyses stratified by obesity status, comorbidities, and medication use may help identify patient populations most likely to benefit from vitamin D supplementation.

Personalized treatment strategies are key future directions. Developing predictive models by integrating clinical characteristics, biomarkers, and VDR gene polymorphisms to identify subgroups that would benefit from supplementation, thereby facilitating the development of personalized therapeutic strategies. However, these genetic findings remain preliminary, and their clinical utility for patient stratification requires prospective validation in well‐powered trials before they can be translated into practice. A 2025 meta‐analysis confirmed that VDR polymorphisms (*Fok*I, *Taq*I, and *Bsm*I) are susceptibility markers for CU, each conferring a 1.5‐ to 2.1‐fold increased risk, but translating these findings into clinical practice needs further validation [46].

Standardization of clinical research is needed. Future randomized, placebo‐controlled trials should stratify by baseline vitamin D levels to define optimal supplementation thresholds, doses, and durations. Key outcomes should include UAS7, DLQI, relapse rates, antihistamine dose reduction, and long‐term safety endpoints (such as hypercalcemia, nephrocalcinosis, and long‐term bone density), rather than relying solely on biochemical parameters.

Combination therapy appears promising. Patients receiving vitamin D alongside other treatments tend to show greater improvements than those on monotherapy [31]. However, most studies should be interpreted with caution given the small sample sizes, short follow‐up periods, and lack of standardized outcome measures in most studies. In addition, the synergistic effects of vitamin D with existing treatments (such as antihistamines, omalizumab, and immunosuppressants) warrant further investigation through well‐designed, adequately powered trials.

Long‐term efficacy and safety data are crucial for guiding clinical practice. The available clinical studies are generally limited in duration, making it difficult to draw firm conclusions regarding the long‐term risks and benefits of high‐dose vitamin D supplementation. Standardized monitoring protocols must be established to evaluate the effects of long‐term high‐dose vitamin D supplementation on calcium and phosphorus metabolism, as well as liver and kidney function. Regular assessment of serum 25(OH)D, calcium, phosphorus, parathyroid hormone, and urinary calcium levels would facilitate early detection and management of potential adverse effects.

Updating clinical guidelines requires evaluating the strengths and limitations of the available evidence. According to the EAACI/GA^2^LEN/EuroGuiDerm/APAAACI guideline, first‐line therapy remains second‐generation H1‐antihistamines, with dose escalation, omalizumab, or cyclosporine as subsequent options [47]. Vitamin D supplementation is not currently recommended as a standard treatment for CU; it is only mentioned as a potential adjunctive therapy requiring further study. Its role remains investigational, and supplementation is advised only for patients with confirmed deficiency based on general health principles, not as a disease‐specific intervention. High‐quality evidence is needed to clarify screening criteria, target levels, and monitoring frequency. Until such evidence emerges, clinicians should weigh the potential benefits against risks based on individual patient circumstances and make personalized decisions. Key evidence gaps that must be addressed before vitamin D can be incorporated into standard CU management include (1) lack of large‐scale, long‐term RCTs with adequate statistical power; (2) unresolved causality (whether deficiency is a cause, consequence, or bystander); (3) absence of validated biomarkers or genetic predictors for patient selection; and (4) undefined optimal dosing, target levels, and treatment duration.

In summary, research on the relationship between vitamin D and CU has made significant progress but still faces numerous challenges. For clinicians, based on current evidence, it is reasonable to consider vitamin D supplementation for CU patients with vitamin D deficiency; however, this should be guided by the principle of individualization, with close monitoring of efficacy and safety.

## 7. Conclusion

Research on the relationship between vitamin D and CU represents a significant advancement at the intersection of dermatology and nutritional immunology. Epidemiological studies consistently demonstrate an association between low vitamin D levels and CU, and mechanistic studies offer plausible biological pathways through which vitamin D may modulate mast cell function, Treg differentiation, and immune homeostasis. The threshold model we propose provides a theoretical framework for understanding this complex relationship, but it remains theoretical and needs validation. At present, the evidence does not support routine vitamin D supplementation as a disease‐modifying therapy for CU. High‐quality RCTs are needed to confirm efficacy, define optimal dosing, and identify suitable patient subsets. In the meantime, clinicians may consider individualized supplementation for patients with CU who are vitamin D deficient, with close monitoring of both clinical response and safety.

## Funding

No funding was received for this manuscript.

## Conflicts of Interest

The authors declare no conflicts of interest.

## Data Availability

Data sharing is not applicable to this article as no datasets were generated or analyzed during this narrative review.
